# Inhibition of BAMBI reduces the viability and motility of colon cancer via activating TGF-β/Smad pathway *in vitro* and *in vivo*

**DOI:** 10.3892/ol.2021.12505

**Published:** 2021-02-02

**Authors:** Wenzhen Yu, Hong Chai

Oncol Lett 14: 4793-4799, 2017; DOI: 10.3892/ol.2017.6811

Following the publication of this paper, it was drawn to the authors’ attention by an interested reader that certain tumour images featured in Fig. 4A of the above paper had apparently been copied and re-inserted within the Figure itself. Furthermore, other features associated with the tumour images presented in this Figure bore strong similarities with tumour images in a number of other figures published in different Journals by different authors from different institutions.

The Editor asked the authors for an explanation to account for the appearance of strikingly similar data in their paper independently; however, our enquiries failed to elicit any reply from the authors. Therefore, the Editor has decided to retract this paper from *Oncology Letters* based on the serious anomalies identified in Fig. 4 without having received a response from the authors. The Editor apologizes to the readership for any inconvenience caused.

